# Improved Visualization of the Necrotic Zone after Microwave Ablation Using Computed Tomography Volume Perfusion in an *In Vivo* Porcine Model

**DOI:** 10.1038/s41598-019-55026-9

**Published:** 2019-12-06

**Authors:** Keno K. Bressem, Janis L. Vahldiek, Christoph Erxleben, Seyd Shnayien, Franz Poch, Beatrice Geyer, Kai S. Lehmann, B. Hamm, Stefan M. Niehues

**Affiliations:** 1 Department of Radiology, Charité, Hindenburgdamm 30, 12203 Berlin, Germany; 2 Department of Surgery, Charité, Hindenburgdamm 30, 12203 Berlin, Germany

**Keywords:** Liver cancer, Liver cancer, Targeted therapies

## Abstract

After hepatic microwave ablation, the differentiation between fully necrotic and persistent vital tissue through contrast enhanced CT remains a clinical challenge. Therefore, there is a need to evaluate new imaging modalities, such as CT perfusion (CTP) to improve the visualization of coagulation necrosis. MWA and CTP were prospectively performed in five healthy pigs. After the procedure, the pigs were euthanized, and the livers explanted. Orthogonal histological slices of the ablations were stained with a vital stain, digitalized and the necrotic core was segmented. CTP maps were calculated using a dual-input deconvolution algorithm. The segmented necrotic zones were overlaid on the DICOM images to calculate the accuracy of depiction by CECT/CTP compared to the histological reference standard. A receiver operating characteristic analysis was performed to determine the agreement/true positive rate and disagreement/false discovery rate between CECT/CTP and histology. Standard CECT showed a true positive rate of 81% and a false discovery rate of 52% for display of the coagulation necrosis. Using CTP, delineation of the coagulation necrosis could be improved significantly through the display of hepatic blood volume and hepatic arterial blood flow (p < 0.001). The ratios of true positive rate/false discovery rate were 89%/25% and 90%/50% respectively. Other parameter maps showed an inferior performance compared to CECT.

## Introduction

Microwave ablation (MWA) is a thermoablative method for minimally invasive tumor treatment^1^. During the procedure, high temperatures are generated around the tip of an antenna positioned in the target tissue, causing a locally limited coagulation necrosis of the tissue^1,2^. MWA can be performed under CT guidance for exact positioning of the antenna in the target tissue with immediate post-ablation contrast-enhanced computed tomography (CECT) to ensure a sufficient ablation of the target tissue. Although CECT has proven to be able to visualize ablation margins after thermoablation in studies^3–5^, a risk of local recurrence remains, even if a tumor appears to be fully ablated. This is due to the fact that only the inner parts of the ablated target area become fully necrotic, while vital tissue may persist in the outer zone, as shown by pathologic and histologic analyses^6–9^. Contrast-enhanced CT alone cannot accurately display the margins of the coagulation necrosis within an ablation^9–11^. An imaging technique allowing for an exact visualization of the coagulation necrosis and early detection of incomplete tumor coverage could ensure and allow prompt re-ablation and thus possibly lower the rate of tumor recurrence.

Previous research suggests, that CT-perfusion (CTP) might improve visualization of ablated areas after thermoablation^12,13^, but, so far, no sufficient analysis has been performed. Therefore, the present study aims to evaluate the accuracy of CTP to visualize the coagulation necrosis after MWA, proposing thresholds as partial guidance for an improved delineation of the necrotic area.

## Results

Overall, 10 ablations were evaluated. Histologically, the mean necrotic area size was 204.8 mm² with a mean maximum diameter of 18.1 mm. The best correlations with histology could be shown for measurements taken in perfusion maps of HBV and AF, which sowed correlation coefficients of r = 0.73 and r = 0.72, both being superior to CECT measurements, respectively. The mean size of the coagulation necrosis was 199.2 mm² for HBV with a mean maximum diameter of 17.5 mm, which represented an underestimation of the true coagulation necrosis extend, as defined by histology. For AF, the mean size was 308.7 mm² with a mean diameter of 22.3 mm, which corresponded to overestimation of the true size. However, compared to the histologic reference standard, these differences were not statistically significant (p = 0.535 for AF and p = 0.083 for HBV). The mean squared error, as a measurement of deviations between CT measurements and histology, with higher values corresponding to a poorer fit to the reference standard, was lowest for HBV and second lowest for AF (97 for HBV and 148 for AF). The measurements from CECT-images showed an overall good correlation with histology (r = 0.59), but also an overestimation of the ablations true size with a measured mean coagulation necrosis of 334.4 mm², corresponding to a significant overestimation (p = 0.038). The mean squared error of size measurements in CECT was 169.

Other perfusion maps showed a lower suitability for visualizing the extent of the coagulation necrosis with an inferior performance compared to the CECT. Although a higher correlation with the histologic reference standard could be observed for PF, TF and MTT with r = 0.7, r = 0.68 and r = 0.65, respectively, all of these parameters showed a significant overestimation of the coagulation necrosis extend (p < 0.05) and larger mean squared errors than CECT. A detailed overview of measured ablation sizes is provided by Table 1.

**Table 1:** Size comparison of contrast enhanced CT and CT-perfusion against the histologic reference standard.

	Mean Area (mm²)	Mean maximum diameter (mm)	p	Correlation coefficient	Mean squared error
Histology	204.8	18.1			
Contrast enhanced CT	334.4	21.0	0.038	0.59	169
CT Perfusion					
Hepatic blood volume	199.2	17.5	0.535	0.73	97
Hepatic arterial blood flow	308.0	22.3	0.083	0.72	148
Portal blood flow	359.8	24.0	0.012	0.70	178
Total blood flow	365.5	23.7	0.017	0.68	181
Mean transit time	356.3	23.7	0.009	0.65	172

Table 1 provides the mean size in mm² of the coagulation necrosis as measured in the histologic reference standard, contrast enhanced CT and the various CT perfusion maps. It shows that hepatic blood volume is the most accurate to display coagulation necrosis compared to histology. The p-value for the t-test for comparison of the size of coagulation necrosis between the histologic reference standard and parameter maps is given in the second row, showing a significant overestimation for contrast enhanced CT, portal venous flow and mean transit time. Correlation of the measured size with the reference standard is given in the third row and the mean squared error between size measurements in histology and contrast enhanced CT or CT perfusion can be obtained from the last row.

### Diagnostic accuracy

**Figure 1:**
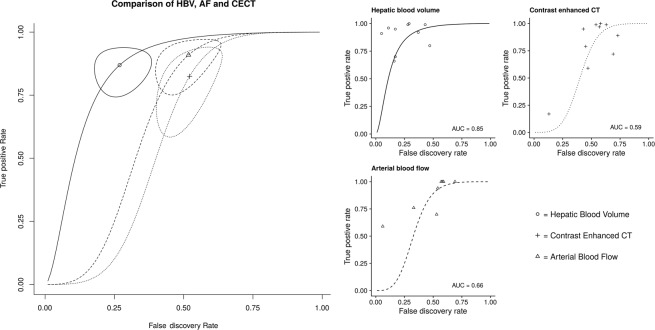
Shows summary receiver operator characteristic (SROC) curves for the accuracy of hepatic blood volume (HBV), hepatic arterial blood flow (AF) and contrast enhanced computed tomography (CECT). On the left, the SROC curves of all three parameter maps with the summary estimate for the overall accuracy and its 95% confidence interval is provided. The three subplots on the right represent the individual SROC curves, and the points represent pairs of true positive and false discovery rates in individual ablations. HBV provides the highest accuracy with an area under the SROC-curve (AUC) of 0.85, followed by AF and CECT. Also, the 95% CI of the summary estimate for HBV does not overlap with the 95% CI for AF or CECT. Between the 95% CI of AF and CECT some overlap can be seen, which could indicate that AF might not perform better than CECT in a different sample.

Accuracies for CECT and the different perfusion maps, subdivided by individual ablated areas, are provided by Table 2. Overall, perfusion maps of AF and HBV, both showing a decrease of values in the dead tissue, were significantly superior for the visualization of coagulation necrosis compared to CECT (p < 0.001). A summary ROC-curve for AF, HBV and CECT is provided by Fig. 1.

**Table 2:** Individual measurements of accuracy for contrast enhanced CT and CT-perfusion.

	Contrast enhanced CT			Arterial blood flow			Portal blood flow			Total blood flow			Hepatic blood volume			Mean transit time		
	TPR (%)	FDR (%)	ADR	TPR (%)	FDR (%)	ADR	TPR (%)	FDR (%)	ADR	TPR (%)	FDR (%)	ADR	TPR (%)	FDR (%)	ADR	TPR (%)	FDR (%)	ADR
Ablation 1	99	54	0.83	100	57	0.76	100	59	0.71	100	53	0.89	100	29	2.42	100	59	0.7
Ablation 2	99	63	0.59	100	59	0.7	100	65	0.54	100	48	1.1	91	5	6.58	99	68	0.47
Ablation 3	59	47	0.63	76	33	1.23	100	48	1.07	100	39	1.57	96	11	5.9	100	49	1.06
Ablation 4	72	69	0.38	7	53	0.65	95	68	0.45	100	69	0.45	80	47	0.87	100	69	0.45
Ablation 5	17	13	0.19	59	06	1.34	100	80	0.25	100	80	0.25	99	28	2.54	100	80	0.25
Ablation 6	95	43	1.27	100	58	0.73	099	58	0.72	100	58	0.73	99	43	1.32	98	49	1.02
Ablation 7	100	58	0.73	100	58	0.73	100	58	0.73	100	58	0.73	95	17	3.97	100	55	0.83
Ablation 8	89	73	0.36	100	69	0.46	100	71	0.41	100	71	0.41	92	37	1.47	100	68	0.48
Ablation 9	79	45	0.92	94	54	0.8	80	67	0.44	100	70	0.42	70	17	1.57	100	67	0.48
Ablation 10	97	57	0.74	100	57	0.75	100	58	0.73	100	58	0.73	66	16	1.42	95	57	0.73
Pooled	**81**	**52**	**0.66**	**90**	**50**	**0.82**	**97**	**63**	**0.6**	**100**	**6**	**0.73**	**89**	**25**	**2.81**	**99**	**62**	**0.65**
Cut-off	200 HU			74 ml/min			75 ml/min			100 ml/min			29 ml			38 sec

The true positive rate/false discovery rate of HBV was 89%/25%, using an upper threshold of 29 ml. The true positive rate indicates, that 89% of the necrotic zone was displayed correctly by HBV and the false discovery rate indicates that 25% of the area displayed by the HBV as necrotic, did not correspond to necrotic tissue in the histologic reference standard, i.e. was displayed incorrectly by HBV. The overall agreement/disagreement ratio off HBV was 2.81. An agreement/disagreement ratio of 1 would indicate that the sum of the true positive pixels is equal to the sum of the false positive and false negative pixels. Accordingly, if the ratio is less than 1, the sum of false positive and false negative pixels is larger than the sum of true positive pixel, and if the ratio is greater than 1, the sum of true positive pixels is larger.

For AF, when an upper threshold of 74 ml/min was used, necrotic areas in the ablated zone were identified with a true positive rate/false discovery rate ratio of 90%/50% and an agreement/disagreement ratio of 0.82. CECT had lower accuracy than both HBV and AF, but higher accuracy than the other perfusion maps (PF, TF and MTT). Using an upper threshold of 200 HU, an agreement/disagreement ratio of 0.66 could be achieved with a true positive ratio of 81% and a false discovery rate of 52%.

For portal venous blood flow and total hepatic blood flow, which also showed decreased flow rates in the centre of the ablations, an even higher agreement in the visualization of necrotic zones could be reached with true positive rates of 97% and 100% respectively, however, this was achieved at the expense of a higher false discovery rate of 63% and 60% and consequently lower agreement/disagreement ratios of 60% and 73% respectively. Mean transit time (MTT) was decreased in the centre of ablation. For an upper threshold of 38 s, a true positive rate/false discovery rate ratio of 99%/62% could be achieved with an agreement/disagreement ratio of 0.65.

Table 2 provides the individual measurements of accuracy for contrast enhanced CT and the various CT perfusion maps. It shows that arterial blood flow and hepatic blood volume both allow for a more accurate depiction of the coagulation necrosis than CECT. For each modality the true positive rate (TPR, higher equals better), false detection rate (FDR, lower equals better) and agreement-disagreement ratio (ADR, higher equals better) are listed. The pooled accuracies are given under the respective individual values. Finally, in the last row, the cut-off used to calculate the accuracies for all ablations are given. HU = Hounsfield units.

## Discussion

The results of the present study show that CTP-parameter maps of arterial blood flow and hepatic blood volume allow for an improved visualization of the coagulation necrosis immediately after ablation in a porcine model of MWA. In clinical practice, this could enable earlier detection of incomplete ablations and thus a timely re-ablation in the same session, potentially reducing the number of local recurrences. The current study also shows that the results of CT perfusion were objectifiable across the animals, as it was possible to establish common thresholds for perfusion values. This suggests that similar thresholds could also be established for humans, and thus improve accuracy of post-procedural imaging. Still, the relatively small sample should also be considered when interpreting these results, especially for AF. As can be seen in Fig. 1, the confidence intervals for the summary estimate overlapped between AF and CECT, which may be an indication that, given a different sample size, the AF might no longer perform better than CECT. It should also be considered that CECT and AF showed overestimation of the coagulation necrosis’ size, which may result in erroneously assessing the ablation size as sufficient, while tumour still persists in the transitional zone.

The CT-based visualization of the central necrotic zone following thermoablation was not explicitly studied previously, as the primary focus of earlier studies was on the identification of ablation margins, overall size assessment or cross-modality comparison of CT to ultrasound, elastography or positron emission tomography (PET)^3,10,14–19^. However, since vital tissue still exists in outer ablation zones and the boundaries of the necrotic areas cannot be reliably extrapolated from the outer shape of the ablation^6,7,20^, the delineation of the outer ablation margins alone is no accurate measure of the success of ablation^9^. Although, as in surgery, a safety zone of 5 mm is recommended for MWA, local recurrence rates are reported to be comparatively higher after ablation^21–23^. Ringe *et al*. showed that the technical success of MWA correlated poorly with the local recurrence rate^24^. So far, magnetic resonance imaging (MRI) appears to be the most accurate modality for assessing the outcome of MWA^24–26^. However, immediate control of ablation using MRI would require either the transfer of the patient from the intervention site to the MRI facility, precluding immediate on-site re-ablation if residual tumor tissue is detected in the target area, or the on-site performance of MRI-guided MWA. For the latter, however, MRI-suitable materials are needed, and so far, most available devices are not MRI safe. However, if MRI-suitable materials can be used novel studies showed that MRI can accurately display the ablation zones and could also be used as real-time monitoring^27,28^. Still, accuracy of needle placement can be hampered in some MRI systems by the comparably narrow gantry, unless an open MRI system is used^29,30^. For these reasons, CTP could shorten and simplify post-ablational imaging procedures, as there is no need to switch from CT to MRI and early re-ablation can be performed on site easily, time- and cost-efficiently. However, this time gain in post-ablational imaging comes at the expense of additional radiation exposure and contrast agent administration, especially if the first ablation proves to be insufficient and re-ablation with subsequent re-imaging is required.

Our study has limitations: Due to the methodology of comparing images of ablations with histological sections, a small disagreement, due to the fixation process of the tissue, can never be avoided possibly degrading the results we obtained for the performance of CECT/CTP. Also, ablations were performed in healthy pigs. The pig liver differs from the human liver in that it contains more interlobular connective tissue, which could affect heat distribution and thus the appearance of the coagulation necrosis^31^. Furthermore, we performed ablations in tumor-free livers. Hence, the findings of this work cannot be fully translated to the ablation and perfusion behaviour of (ablated) tumor tissue, as heat dispersion and development of necrosis may differ in tumor tissue. In addition, it was not possible to evaluate whether tumor tissue would have remained in the transitional zone after an ablation, even though the extent of the ablations seemed sufficient after perfusion imaging in healthy liver tissue. A further limitation is, that a single cut-off was selected for each perfusion map and the CECT-images to calculate the accuracies, as this is the only way to generalize the results. However, the generalized cut-off differs from the ideal cut-off for each individual ablation. Finally, the use of shuttle perfusion may also contribute to a decrease in CTP accuracy, since continuous bi-directional table movement causes temporal shifts in the recording of the same contrast medium phase at the upper and lower edge of the examined region. Short contrast-phases, such as maximum arterial enhancement, could be blurred, and the motion and time corrections performed during pre-processing are likely limited. During data analysis, we also observed that it was difficult to establish a cut-off for ablations directly adjacent to the diaphragm, because the algorithm used for image segmentation did not recognize ablation boundaries when perfusion and/or attenuation values in the adjacent diaphragm or lungs were too low. However, this should not cause any problems for a human observer, as these structures are easy to recognize in CT images and/or overlaid perfusion maps. Nevertheless, it is still advisable to maintain a sufficiently large safety margin during ablation. Finally, it has been shown, that the inner portions of the transitional zone deteriorate in the first 12 hours after ablation, indicating a further expansion of the coagulation necrosis^32^. due to the immediate explantation of the pig livers, in our experiment, histology might have underestimated the true extent of final coagulation necrosis.

Overall, the here presented approach for visualizing post interventional coagulation necrosis using CTP allowed for an improved delineation of the necrotic area compared to standard CECT.

In conclusion, CT-perfusion is superior to CECT in the delineation of the necrotic tissue after hepatic microwave ablation, particularly when assessment is based in the visualization of the reduced hepatic blood volume and arterial perfusion of ablations.

## Materials and Methods

### Animals, housing and care

This prospective animal study was approved beforehand by the National Office for Health and Social Affairs as well as by the institutional review board of Charité Universitätsmedizin Berlin and conducted according to the guidelines and rules of the Federation of Laboratory Animal Science Associations. The animals were kept under controlled conditions in our department’s animal facilities complying with the specifications of local guidelines and the Animal Welfare Act. Microwave ablations and CTP were performed in five domestic pigs. The pigs were anesthetized throughout the procedure and were cared for by an experienced veterinarian.

### Microwave ablation

All ablations were performed under CT guidance using an 80-Slice CT-scanner (Aquilion Prime, Canon Medical Systems Corporation, Japan). Before placement of the antennas (AveCure, MedWaves Incorporated, San Diego, USA), the liver was exposed by median laparotomy to ensure more precise needle positioning. Each ablation was performed with a power of 100 W, delivering a target energy of 24 kJ into the tissue and followed by CTP. Before needle retraction, a 2-cm plastic tube was inserted centrally into the ablation, enabling reliable re-location of the ablation axis and histological sectioning orthogonally to the axis in the ablation center. Between one and two ablations with subsequent CTP were performed per animal.

### Histological sections

The preparation of histological slices was performed by an experienced veterinarian who was supervised by a veterinary pathologist. After liver explantation, ablations were resected in toto and cut orthogonally through the axis, as indicated by the plastic tube. A specifically designed template was used for accurate cutting^33^. Ablations were embedded in Tissue-Tek-O.C.T.-Compound (Sakura-Finetek-Germany GmbH, Germany) and deep-frozen. Histological sections were obtained with a cryostat (CryoStar-NX70-Cryostat, Thermo-Fischer-Scientific, USA) and mounted on glass slides. Thereafter, sections were stained according to a protocol of Neumann *et al*. using a Hematoxylin & Eosin and a nicotinamide adenine dinucleotide (NADH) and nitro blue tetrazolium chloride (NBTC) solution^34^. Subsequently, sections were photographed with a digital microscope (BZ-X810, Keyence-Cooperation, Japan) and stitched using the ImageJ-2D/3D-Stitching-Plugin^35,36^. The zone of coagulation necrosis was semi-automatically segmented with the fuzzy-selection tool of the GNU-Image-Manipulation-Program (The-GIMP-Team, GIMP2.8.10, www.gimp.org, 1997–2018) and exported in the standard Digital Imaging and Communications in Medicine (DICOM) format.

### CT-Perfusion

The same scanner was used for MW-needle positioning and CTP. Scan parameters were: 100 kV, 200 mA, 0.35 s rotation time and 1 mm reconstructed slice thickness. For full liver coverage, CTP was performed in shuttle mode with constant bidirectional table movement covering an area of 16 cm, with the acquisition of 40 CT volumes over the course of 3 seconds for each volume. During CTP, 40 ml iomeprol (Imeron 400, Bracco Imaging, Italy) were injected at 5 ml/s. Thereafter, images were co-registered with a non-rigid algorithm to reduce motion artifacts and, because of shuttle perfusion, time correction was applied. Images were reconstructed using a FC11 convolution kernel.

### Contrast Enhanced CT

Contrast enhanced CT was performed after CTP and after injection of 100 ml iomeprol (Imeron 400, Bracco Imaging, Italy) at a speed of 4 ml/s. Scan parameters for CECT were 120 kV, 400 mA, 0,5 s rotation time and 0,5 mm slice thickness with FC 11 convolution kernel.

The “Research PC”, software version V6.0SP0500EWiP, (Canon Medical Systems Corporation, Japan) was used for further data readout. For all evaluations, a hepatic dual input deconvolution algorithm with a box-like impulse residue function was applied, allowing calculation of hepatic blood volume (HBV), hepatic arterial blood flow (AF), portal venous blood flow (PF), total hepatic blood flow (TF) and mean transit time (MTT). Finally, multiplanar reconstructions of CECT and CTP-scans (corresponding to the histological sections) were exported in DICOM format.

### Data analysis

**Figure 2:**
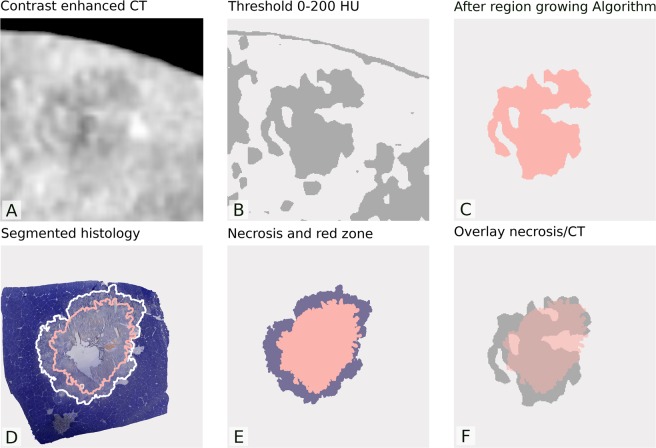
Schematically shows the image analysis procedure. First the images (in this case CECT) were imported (1A), then grey level mapping was applied, showing only values between 0 and a given cut-off value, transforming the image into a binary image (1B). The ablation was then isolated by a region-growing algorithm (1C).

**Figure 3:**
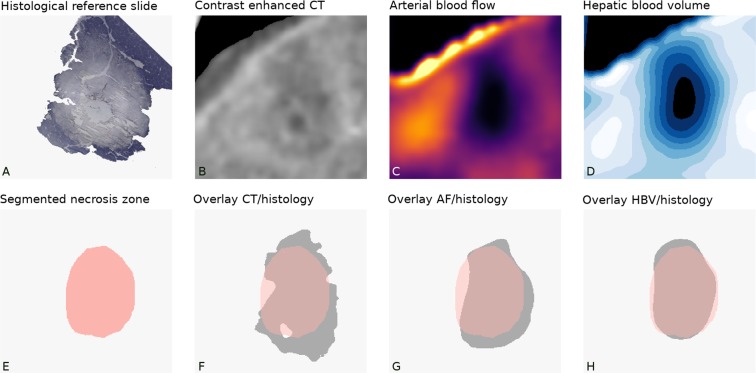
Shows a comparison in display of the coagulation necrosis of histology (Hematoxylin and eosin stain) (2A), contrast enhanced CT (2B) and perfusion maps of hepatic arterial flow (2C) and hepatic blood volume (2D). Under the respective original images, the segmented coagulation necrosis of the gold standard is shown in light red (2E) and the display of the coagulation necrosis by the corresponding modality in dark grey (2F for Contrast enhanced CT, 2G for hepatic arterial flow, 2H for hepatic blood volume). For each modality, the cut-off described in Table 2 was used.

Data and statistical analysis was performed using the “R” statistical software (Version 3.5.0, 2018) including the “ggplot2” package for graphic display and “oro.dicom” for reading DICOM images^37–39^. Images were scaled to a unified pixel size and imported as two-dimensional arrays. Rescaling of attenuation values was performed using the rescale-slope and intercept stored in the DICOM header. Subsequently, ablations were isolated by first setting an upper threshold for the attenuation or perfusion, then strictly windowing the images according to the predefined threshold to obtain binary image and finally applying a region growing algorithm (see Fig. 2). The isolated ablation was overlaid by the histologic segmentation to count matching pixels (true positives) and non-matching pixels (false positives and false negatives). The overlay procedure and calculation of true positives, false positives and false negatives are illustrated by Fig. 3. To reduce overlay errors, the previously inserted plastic marker was used to avoid shifting between the images^33^. To adjust rotation of the images to each other, adjacent vessels and, in some sections, the nearby liver surface were used. For each CECT and CTP image, this procedure was repeated with thresholds varying between 1 and the maximum of the pixel values in the corresponding image.

In the histological images (liver sections stained with NADH-vital stain), the central part of coagulation necrosis and the transitional zone containing residual vital tissue (also referred to as red-zone) were semi-automatically segmented (1D) and isolated (1E). Finally, the segmented coagulation necrosis was overlaid with the CECT or CTP image to calculate the accuracy. HU = Hounsfield units.

### Statistical analysis

Based on counts of true positives, false positives and false negatives, the true positive rate (which equals the agreement between histologic reference standard and CECT/CTP), false discovery rate (disagreement between CECT/CTP and histologic reference) and agreement-disagreement-ratio (pixel-sum of true positives divided by pixel-sum of false positives and false negatives) were calculated. Using repeated measurements with varying thresholds, we performed a receiver operator characteristic (ROC) analysis to identify the threshold for optimal display of the coagulation necrosis in the corresponding image and across all measurements. Accuracies were compared using the chi-squared test. The overall accuracy was also displayed in summary ROC curves (Fig. 1). Extents of coagulation necrosis were calculated over the absolute pixel number. Size measurements were compared to the histologic reference standard using student’s t-test, Pearson’s correlation coefficient and the mean squared error. A p-value < 0.05 was considered statistically significant.

## Data Availability

The datasets generated and/or analysed during the current study are available from the corresponding author upon reasonable request.
